# Multimodal dataset for indoor 3D drone tracking

**DOI:** 10.1038/s41597-025-04521-y

**Published:** 2025-02-12

**Authors:** Jakub Rosner, Tomasz Krzeszowski, Adam Świtoński, Henryk Josiński, Wojciech Lindenheim-Locher, Michał Zieliński, Grzegorz Paleta, Marcin Paszkuta, Konrad Wojciechowski

**Affiliations:** 1 Polish-Japanese Academy of Information Technology, ul. Koszykowa 86, 02-008 Warsaw, Poland; 2 Faculty of Electrical and Computer Engineering, Rzeszow University of Technology, al. Powstancow Warszawy 12, 35-959 Rzeszow, Poland; 3 Department of Graphics, Computer Vision and Digital Systems, Silesian University of Technology, Akademicka 16, 44-100 Gliwice, Poland

**Keywords:** Computer science, Applied mathematics

## Abstract

The subject of the paper is a multimodal dataset (DPJAIT) containing drone flights prepared in two variants – simulation-based and with real measurements captured by the gold standard Vicon system. It contains video sequences registered by the synchronized and calibrated multicamera set as well as reference 3D drone positions in successive time instants obtained from simulation procedure or using the motion capture technique. Moreover, there are scenarios with ArUco markers in the scene with known 3D positions and RGB cameras mounted on drones for which internal parameters are given. Three applications of 3D tracking are demonstrated. They are based on the overdetermined set of linear equations describing camera projection, particle swarm optimization, and the determination of the extrinsic matrix of the camera attached to the drone utilizing recognized ArUco markers.

## Background & Summary

Indoor tracking aims to determine the positions of an object moving inside the building in successive time instants. The simplified variant – 2D tracking – operates only in two dimensions, usually not taking into consideration the altitude value specified in relation to the ground level^1,2^. It is insufficient in the case of flying objects, for which all three dimensions of 3D position are of the same importance. In the full 3D tracking challenge, 3D coordinates in the global system are established based on the measurements provided by attached sensors or a vision system. Due to a limited or even complete lack of GPS signal, the most broadly used RTK GPS (Real Time Kinematic Global Positioning System) is unworkable for indoor applications. The most promising is a visual inspection that registers calibrated and synchronized video sequences from different viewpoints. Based on the object detection as well as the triangulation technique, it is feasible to reconstruct the 3D position.

There are plenty of methods used for the purpose of object tracking. In^3^ Nguyen *et al*. proposed a method for indoor 2D object detection and tracking in short-range using impulse-radio ultra-wideband (IR-UWB) technology. This approach allowed for obtaining quite accurate results in 2D object detection and tracking. However, as was noted by the authors, to improve the results, more sensors are needed. This, combined with algorithm development, could allow tracking objects in 3D by determining their exact position in space. The example of 3D multiple object detection and tracking (MODT) algorithm for autonomous driving tasks was shown in^4^ where authors proposed utilizing LiDAR sensors for that purpose. To optimize the calculations, the sensor signal was merged and processed by an algorithm using an Unscented Kalman Filter and Bayesian filters to determine the positions of detected objects over time. Another approach for 3D object tracking in the case of autonomous vehicles was presented by Yin *et al*.^5^ where point-clouds represent the objects. For this, the authors used a two-stage detector named CenterPoint. The first stage determines the object’s centers using CenterNet detector^6^ and the second one reproduces the geometric structure performing regression and providing superior object detection and tracking results. A common feature of the mentioned algorithms is the need for complex datasets to train the model, as well as to validate its performance.

In recent years, the development of technology has made drones increasingly popular and accessible to everyone. This causes the need to create algorithms dedicated to 3D drone detection and tracking for precise navigation through complex environments, ensuring safe flights and obstacle avoidance, making them more user-friendly. One of the areas where 3D drone tracking algorithms play an important role are augmented reality games^7^. These are gaining popularity around the world by holding competitions where players have the chance to race drones around virtual tracks. Algorithms of this type have also found application in industry^8,9^ by enabling efficient data collection and analysis. In addition, 3D drone tracking is also important for security reasons^10^. The popularity of drones means that they can, for example, pose a threat to air traffic or be used to obtain sensitive information. Therefore, to counter such situations it is important to develop more accurate 3D drone tracking methods. However, this task is quite challenging due to the small size of drones, as well as their ability to move quickly, so it is important to produce complex datasets that can enable development in this area. Table 1 provides a brief description of the published datasets as well as characteristics of our proposed DPJAIT dataset.

**Table 1:** Comparison of datasets (meaning of the superscripts used: *a* - one of the sequences comes from another dataset, *b* - static cameras, *c* - FPV camera, *d* - frame rate of Snapdragon Flight/miniDAVIS346 event camera, respectively, *r* - real data, *s* - simulated data).

	UZH-FPV^11^	EuRoC^28^	MVDT^12^	Mid-Air^15^	Blackbird^16^	UPenn^17^	DPJAIT
# of sequences	31	11	5^*a*^	54	186	4	31 (18^*r*^, 13^*s*^)
Sequence type	real	real	real	sim	real	real	real & sim
Duration [min]	23	22	24 (4 seq.)	79	over 600	—	66 (46^*r*^, 20^*s*^)
Max. dist. [m]	333.6 indoor 735.5 outdoor	130.9	no data	no data	860.8	700	669
# of drones	1	1	1	1	1	1	1-10
Static cameras	no	no	4-7	no	no	no	4^*r*^, 8^*s*^
FPV cameras	yes	yes	no	yes	yes	yes	yes
Video res. [px]	640 × 480	752 × 480	1440 × 1080 1920 × 1080 3840 × 2160	1024 × 1024	1024 × 768	960 × 800	1924 × 1082^*b*^ 3840 × 2160*c*
Video freq. [fps]	30/50^*d*^	20	25-60	25	120	40	25
IMU [Hz]	500/1000	200	—	100	100	200	—
Ref. data system	Leica	Leica/Vicon	GNSS	n/a	mocap	no data	Vicon
Ref. data [Hz]	20	20/100	100	n/a	360	no data	100
ArUco markers	no	no	no	no	no	no	yes

The UZH-FPV Drone Racing dataset^11^ contains the First Person View (FPV) data obtained during the aggressive high-speed flight of the drone controlled by an expert pilot. Datasets include the recordings from the pilot’s high-resolution FPV camera and miniDAVIS346 event camera as well as Inertial Measurement Unit (IMU) signals. Images and IMU data were recorded to ROS bag files (ROS – Robot Operating System). The ground truth 6DoF trajectories were generated using a Leica Nova MS60 laser tracker. The flight sequences were recorded both indoors and outdoors.

The 11 sequences from the EuRoC (European Robotics Challenge) MAV (Micro Aerial Vehicle) visual-inertial datasets were recorded in two environments: in a large machine hall at ETH Zürich and in a room equipped with a Vicon motion capture system. A visual-inertial sensor unit consisting of two monochrome cameras and an IMU was mounted on an AscTec Firefly MAV and provided, respectively, stereo images (at a rate of 20 Hz) and gyroscope and accelerometer readings (at a rate of 200 Hz). Position ground truth was measured with a Leica Nova MS503 laser tracker, and 6D pose ground truth was recorded using a Vicon system.

The multi-view drone tracking (MVDT) datasets^12^ contain the data captured with multiple consumer-grade (low-cost) cameras from the ground level. The dataset includes highly accurate 3D drone trajectory ground truth recorded by a precise real-time RTK system. The repository contains 5 datasets. Three datasets have temporal synchronization; for two sets, the ground truth camera locations were provided. Four sets contain the 2D labels of drones, but only one includes the 3D orientation.

The quality of graphics offered by modern game engines such as Unreal Engine or Unity, combined with the capabilities of modern GPU graphics systems, increased the popularity and extended application areas of synthetic data. Simulation environments like Microsoft AirSim^13^ could be used to generate virtual sensor data. Some data modalities like depth maps or optical flow could be obtained with a precision unattainable in real conditions. The simulation data can be successfully used to train a semantic segmentation model responsible for terrain classification for potential landing spot detection^14^. The Mid-Air^15^ contains 79 minutes of a drone flight in the virtual environment. In addition to data from the simulated IMU and GPS sensor, the vision data in 1024 × 1024 resolution with an additional modality like dense depth map, stereo disparity map, surface normal map, and semantic segmentation were provided.

The hybrid Blackbird dataset^16^ offers 5 environments with 186 sequences. The dataset contains the 100 Hz real data of IMU and 360 Hz reference ground truth from mocap-based positioning. The sets include sensor data from 120 Hz stereo and downward-facing photorealistic virtual cameras rendered using the game engine based on the read data.

The fast flight dataset from GRASP Laboratory at the University of Pennsylvania, denoted as UPenn^17^, is another example of combining two forward-looking cameras and one IMU sensor in Visual Inertial Odometry (VIO). The dataset contains 4 sequences recorded during 300 m round-trip straight line flight with top speeds of 5 m/s, 10 m/s, 15 m/s, and 17.5 m/s, respectively.

The subject of the paper is the dataset with real and simulated data, focused on the problem of drone indoor 3D tracking using multicamera registration. In the proposed scenarios, drones flying in the hall are captured by RGB cameras as well as by highly precise motion capture acquisition. There are also variants of drones with attached cameras recording the view from their perspective. Thus, the published dataset contains video recordings for 3D reconstruction as well as very accurate reference data. Based on the limitations of current datasets and literature, the main features of the DPJAIT dataset can be identified as follows:

- sequences with multiple drones;
- real and simulated sequences;
- data from static and FPV cameras;
- highly precise reference mocap data;
- sequences with ArUco markers.

Moreover, the proposed drone 3D tracking techniques for Inside-Out and Outside-In variants were successfully validated on the DPJAIT dataset.

## Data Records

**Fig. 1:**
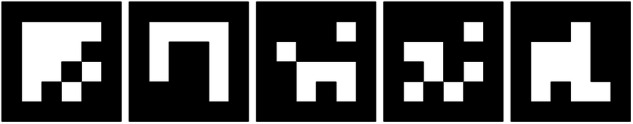
Exemplary ArUco markers with IDs: 8, 10, 36, 39, 45 (from left to right) built on binary matrix 4 × 4.

The dataset can be downloaded from the ZENODO repository^18^. From the top level, the dataset consists of data from both main types of localization systems, namely the Inside-Out type, where the object’s own single or multimodal sensor data are used to estimate its own translation and/or rotation in a known, global coordinate system, and the Outside-In type, where the same is obtained using outside sensor systems able to detect the drone. The former type of data is an FPV stream from a camera mounted motionless on a drone as the built-in gimbal-mounted camera. It does not provide continuous translation and rotation to the drone’s local coordinate system, which is crucial in this case. To unequivocally define the drone’s location, the room’s vertical surfaces are sparsely marked by unique black and white ArUco markers, each with known positions of its corners in a common global coordinate system. An ArUco marker is a synthetic fiducial square image composed of a binary matrix surrounded by a black border (Fig. 1). The internal matrix encodes a unique binary pattern that identifies each marker. The external black border facilitates its fast detection in the image or real environment and the binary pattern as its identifier allows for its unambiguous recognition.

The dataset consists of real measurements registered by a Vicon system containing a synchronized RGB multicamera set and motion capture acquisition, as well as simulated sequences obtained from a similar but virtual camera system created in Unreal Engine and AirSim simulator. The scene for the simulation sequences was prepared using a model of the Human Motion Lab (HML) at the Polish-Japanese Academy of Information Technology in Bytom, Poland, in which real sequences were registered.

**Fig. 2:**
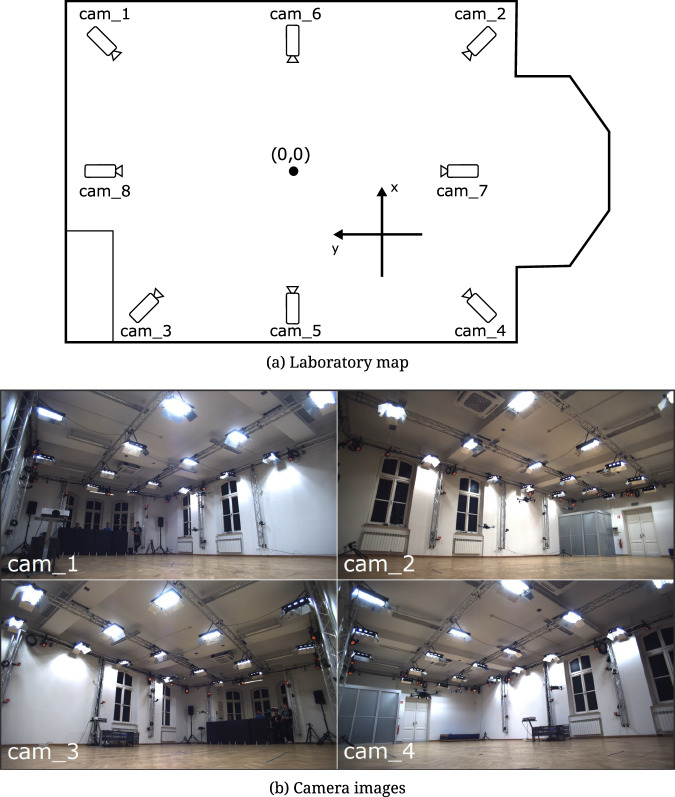
Top view of the laboratory scene with the position of RGB cameras and four camera images from the HML lab.

In the Outside-In variant, drones fly indoors in the lab depicted in Fig. 2. They are registered by an external synchronized and calibrated RGB multicamera set. Thus, there are video frames corresponding to subsequent time instants with the drones. For validation purposes, the 3D positions determined by a reference system are also provided.

When implementing the Inside-Out variant, the before-mentioned method of determining the drone’s position based on the arrangement of fiducial ArUco markers on the walls of the laboratory room in accordance with a previously developed plan was used to generate both real and simulation data.

Sequence calibration was performed using methods from the OpenCV library. The internal parameters of the cameras were taken from the camera and lens specifications and the settings that were entered into the Vicon system. Based on the internal parameters, the 2D positions in the image, and the corresponding 3D positions, the external parameters of the cameras were determined. In the case of real sequences, the 2D positions were obtained by manually marking the markers placed on the drone for selected images of the sequence. For these positions, the corresponding 3D positions were obtained from the C3D files generated by the Vicon system. In terms of simulation sequences, additional scenes with ArUco markers were prepared. The 2D positions of the ArUco markers were detected in the image, and the corresponding 3D positions used were known from a simulation environment. The calibration parameters obtained were saved as CSV files. Distances are given in millimeters, while angular values are in radians.

### Simulated data

The dataset consists of 13 simulation sequences, which differ in the number of drones and their pattern of moving on scene. The sequences were prepared in such a way that they could be used for various types of research. Some sequences contain a larger amount of drones but with limited motion or a smaller amount with a bigger degree of freedom. Additionally, some sequences were generated based on measurements performed in a real laboratory, so they can be used to compare the results obtained for simulation and real sequences.

**Fig. 3:**
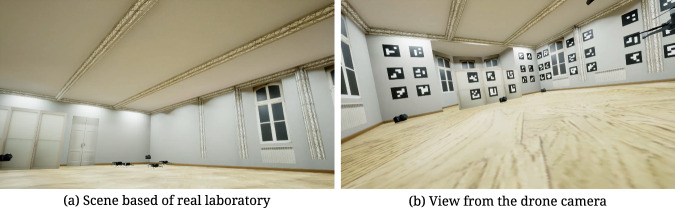
Example photos from Unreal Environment.

The simulation sequences were created using an environment based on the Unreal Engine and the AirSim plugin^13^ (Fig. 3). It is an open-source project created by Microsoft to provide high-fidelity simulation of a variety of autonomous vehicles. Inside the environment, a scene was created based on the laboratory where real-life recordings were performed. For the simulation, eight different cameras were placed; their positions are shown in Fig. 2. For some sequences, the stage size was enlarged to twice the size of the HML laboratory to accommodate more flying drones without an issue of potential collisions between each of them. This allowed for the generation of sequences with a large number of drones (up to 10), which was not possible to achieve in real conditions. Five different drone models were used in the simulations. In addition to the default one provided by AirSim, four unique drone models were implemented. One is based on DJI Mavic, and the other ones are custom constructions. Most sequences contain data from eight cameras (Fig. 2), except three sequences generated based on real sequences (S11_D4, S12_D3, S13_D3), which contain only data from four cameras. In addition, sequences S01_D2_A, S02_D4_A, and S03_D10_A contain images from the drone camera (First Person View, FPV), and ArUco markers placed on walls. In addition to simulations in Unreal Engine, corresponding Python code was developed. It uses the AirSim API to collect event data to generate a dataset and manipulate the simulation at runtime. The first step is to obtain flight paths that will be used to generate the sequences. They can be provided based on recordings from a real environment, using hardcoded trajectories (e.g., sinusoidal), or manually, where a game controller was used. Its position and rotation were recorded in real-time with target frequency and stored in a CSV file. Such a file can be later used to repeat the original path without the need for interaction with a human operator. This allows us to repeat the same experiment multiple times, modifying only some parameters and comparing differences.

In summary, each simulation sequence contains:

- 8 (or 4) video files, each providing the visual output from a single camera, with a frame rate of 25 frames per second (FPS);
- binary masks for each drone;
- time series of drone positions, orientations, and 3D markers coordinates;
- internal and external camera parameters – focal length, principal point as well as position and orientation;
- drone detections using a deep convolutional neural network (YOLOv5^19^);
- coordinates of ArUco markers on the walls (ArUco sequences only).

The summary describing all the simulation sequences of the released dataset is presented in Table 2. The total duration of all of them is 20 minutes and 3 seconds. If multiplied by the number of flying drones, it is 91 minutes and 25 seconds.

**Table 2:** Summary of the simulated sequences.

Seq. ID	# of drones	# of frames	Duration [sec]	Flight pattern	Other
S01_D2_A	2	500	20	free, following	ArUco, FPV
S02_D4_A	4	250	10	free, following	ArUco, FPV
S03_D10_A	10	200	8	sinusoidal, synchronized	ArUco, FPV
S04_D10	10	250	10	free, synchronized	
S05_D8	8	500	20	free, synchronized	
S06_D6	6	500	20	free, synchronized	
S07_D8	8	500	20	sinusoidal, asynchronous	
S08_D8	8	2999	120	free	
S09_D6	6	3000	120	free	small drones
S10_D6	6	3000	120	free	
S11_D4	4	3500	140	free	based on seq. R12_D4
S12_D3	3	9000	360	free	based on seq. R13_D3
S13_D3	3	5875	235	free	based on seq. R14_D3

### Real data

**Fig. 4:**
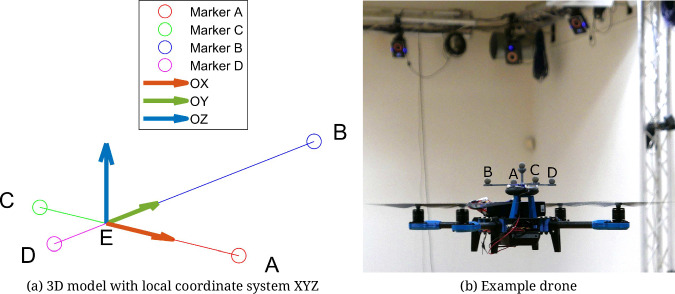
Markers forming asymmetric cross attached to a drone.

In real data scenarios, drones are manually controlled by skilled operators and tracked by a multi-modal acquisition system. Videos are registered by a set of four RGB cameras – cam_1, cam_2, cam_3, and cam_4 – with 1924 × 1082 resolution, located in the corners of the lab, as depicted in Fig. 2. Video frames of randomly chosen time instant and recording are shown in Fig. 2. Moreover, motion capture measurements can be used to provide reference locations and orientations. It can be achieved by tracking four markers – *A*, *B*, *C*, and *D* – attached to the top of the drones and forming an asymmetrical cross, as illustrated in Fig. 4. Details on how to establish the location and orientation in case the known 3D coordinates of the markers are described by Lindenheim-Locher, W. *et al*.^19^. Moreover, to distinguish different drones visible at the same time instant, various lengths of the cross arms are applied. Ground truth data were acquired using a Vicon motion capture system. Synchronization and calibration of the Vicon system and video cameras were carried out automatically according to the Vicon-specified procedure.

In summary, every real data recording consists of:

- four synchronized video sequences registered by cam_1, cam_2, cam_3, and cam_4 cameras;
- time series of 3D markers coordinates of the asymmetrical cross synchronized with video data;
- internal and external camera parameters – focal length, principal point, image distortion coefficients, as well as position and orientation;
- drone detections using a deep convolutional neural network (YOLOv5^19^).

**Fig. 5:**
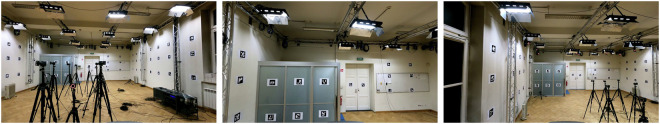
View from the camera attached to drone registering ArUco markers.

As mentioned earlier, there are also registration scenarios for the Inside-Out variant. They are based on the drone tracking of ArUco patterns attached to the walls of the HML lab, as seen in Fig. 5. The 3D coordinates of the ArUco patterns are established in the preliminary stage on the basis of mocap markers placed in their corners and an extra infrared camera set recording them from the center of the lab. In this scenario, the drones are equipped with the GoPro camera mounted at the top. As it is a separate component working outside the Vicon system, manual synchronization is required for the reference mocap data. A detailed description of this operation can be found in the section “Technical validation” (subsection “ArUco markers”).

**Fig. 6:**
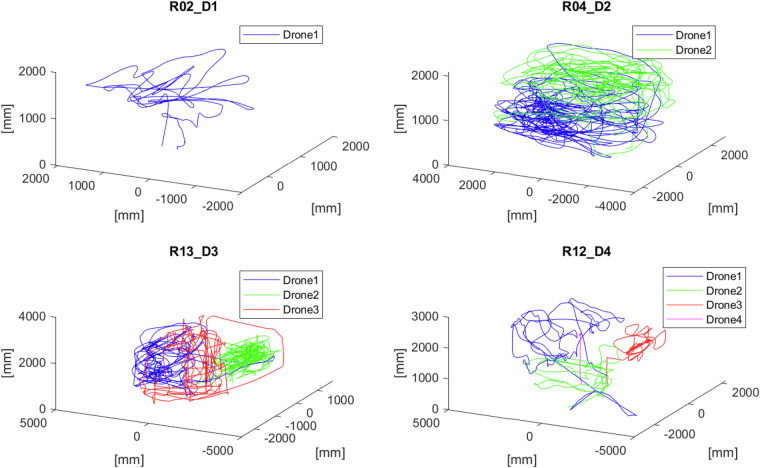
Drone trajectories for randomly selected recordings.

The summary describing all the HML recordings with real measurements of the released dataset is presented in Table 3. The total duration of all of them is 46 minutes and 12 seconds. If multiplied by the number of flying drones, it is 82 minutes and 54 seconds. The example 3D trajectories representing the route of the flying drone are depicted in Fig. 6.

**Table 3:** Summary of the real recordings of the released dataset.

Name	# of drones	# of frames	Duration [sec]	Flight pattern	Other
R01_D2	2	1866	75	free	
R02_D1	1	1514	61	free	
R03_D1	1	1511	60	free	
R04_D2	2	14128	565	free	
R05_D1	1	15327	613	free	
R06_D1	1	2294	92	H pattern	
R07_D1	1	2261	90	free	
R08_D1	1	1526	61	H pattern	
R09_D1	1	570	23	H pattern	
R10_D1	1	2591	104	free	
R11_D1	1	8147	326	free	
R12_D4	4	3500	140	free	corresp. sim. seq. S11_D4
R13_D3	3	9000	360	free	corresp. sim. seq. S12_D3
R14_D3	3	5875	235	free	corresp. sim. seq. S13_D3
R15_D3	3	500	20	in place	
R16_D1_A	1	1932	77	free	ArUco, FPV
R17_D1_A	1	1861	74	free	ArUco, FPV
R18_D1_A	1	1350	54	free	ArUco, FPV

## Methods of 3D localization

The collected dataset is applied to the problem of 3D localization on the basis of RGB registration. It means by analyzing video sequences, 3D coordinates for successive time instants are determined. As previously described, two variants – namely Outside-In and Inside-Out – are taken into consideration.

### Outside-In multicamera system

**Fig. 7:**
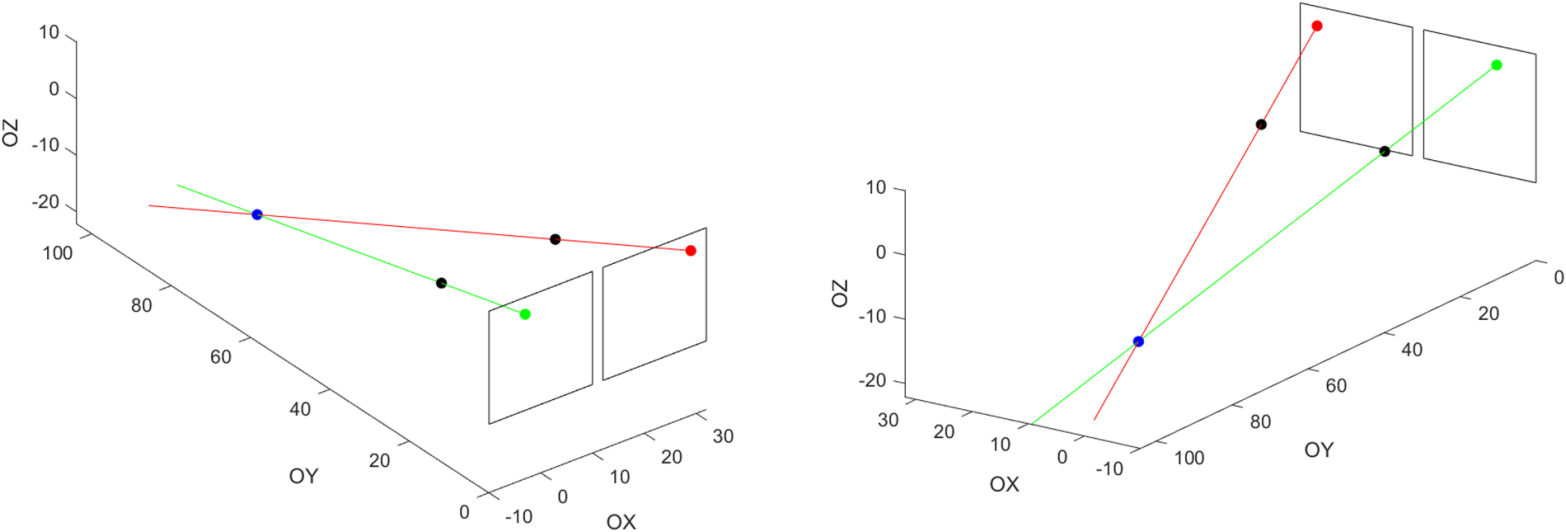
3D reconstruction on the basis of intersection of projection lines denoted by red and green colors. The red/green, blue, and black dots correspond to image points, reconstructed 3D point, and focuses of the camera lens, respectively.

In this scenario, drones are detected on video sequences registered by a set of static and synchronized cameras for which external and internal parameters are known. It can be achieved by motion detection techniques such as background subtraction or more efficiently by neural networks as described by Locher *et al*.^19^. If we know the drone positions on image frames – the centers are taken – and the way in which cameras perform projections, it is feasible to establish the 3D drone coordinates in the global system. Below, two techniques are described for implementing this task. They are based on the determination of the intersection of the projection lines for subsequent cameras as visualized in Fig. 7 and particle swarm optimization.

#### Projection lines

In the case of known internal (intrinsic matrix $$K=\left[\begin{array}{ccc}f & 0 & {c}_{x}\\ 0 & f & {c}_{y}\\ 0 & 0 & 1\end{array}\right]$$) containing focal length *f* and principal point (*c*_*x*_, *c*_*y*_) as well as external camera parameters (rotation matrix *R* and coordinates of the camera in the world system *C*), the projection matrix (camera matrix) *P* can be formed^20^

1 $$P=K[R| t]$$

where *t* = − *R* ⋅ *C*.

It specifies how the projection – the transformation of 3D coordinates [*X*, *Y*, *Z*]^*T*^ to 2D [*x*, *y*]^*T*^ image ones – is performed

2 $$\left[\begin{array}{c}u\\ v\\ w\end{array}\right]=\left[\begin{array}{cccc}{p}_{1,1} & {p}_{1,2} & {p}_{1,3} & {p}_{1,4}\\ {p}_{2,1} & {p}_{2,2} & {p}_{2,3} & {p}_{2,4}\\ {p}_{3,1} & {p}_{3,2} & {p}_{3,3} & {p}_{3,4}\end{array}\right]\cdot \left[\begin{array}{c}X\\ Y\\ Z\\ 1\end{array}\right]$$

where $$x=\frac{u}{w}$$, $$y=\frac{v}{w}$$ and $$P=\left[\begin{array}{cccc}{p}_{1,1} & {p}_{1,2} & {p}_{1,3} & {p}_{1,4}\\ {p}_{2,1} & {p}_{2,2} & {p}_{2,3} & {p}_{2,4}\\ {p}_{3,1} & {p}_{3,2} & {p}_{3,3} & {p}_{3,4}\end{array}\right]$$.

Thus, the 3D coordinates are established on the basis of the accuracy of their projection to image data. The following procedure is used. For every camera, two linear equations with three unknowns *X*, *Y*, *Z* are prepared

3 $$\begin{array}{l}({p}_{1,1}-x\cdot {p}_{3,1})\cdot X+({p}_{1,2}-x\cdot {p}_{3,2})\cdot Y+({p}_{1,3}-x\cdot {p}_{3,3})\cdot Z=x\cdot {p}_{3,4}-{p}_{1,4}\\ ({p}_{2,1}-y\cdot {p}_{3,1})\cdot X+({p}_{2,2}-y\cdot {p}_{3,2})\cdot Y+({p}_{2,3}-y\cdot {p}_{3,3})\cdot Z=y\cdot {p}_{3,4}-{p}_{2,4}\end{array}$$

In total, there are twice the equations as the number of cameras. They can be represented in the matrix form

4 $$L\cdot \left[\begin{array}{c}X\\ Y\\ Z\end{array}\right]=B$$

where for every camera with known projection matrix *P* and image coordinates (*x*, *y*) of the localized drone, there are two rows of the matrix *L* and *B*

5 $$L=\left[\begin{array}{ccc}{p}_{1,1}-x\cdot {p}_{3,1} & {p}_{1,2}-x\cdot {p}_{3,2} & {p}_{1,3}-x\cdot {p}_{3,3}\\ {p}_{2,1}-y\cdot {p}_{3,1} & {p}_{2,2}-y\cdot {p}_{3,2} & {p}_{2,3}-y\cdot {p}_{3,3}\end{array}\right]\,B=\left[\begin{array}{c}x\cdot {p}_{3,4}-{p}_{1,4}\\ y\cdot {p}_{3,4}-{p}_{2,4}\end{array}\right]$$

As a result, the overdetermined system of linear equations is obtained. It can be solved by the product of the pseudoinverse matrix (*pinv*) to *L* and *B*

6 $$\left[\begin{array}{c}X\\ Y\\ Z\end{array}\right]=pinv(L)\cdot B$$

**Fig. 8:**
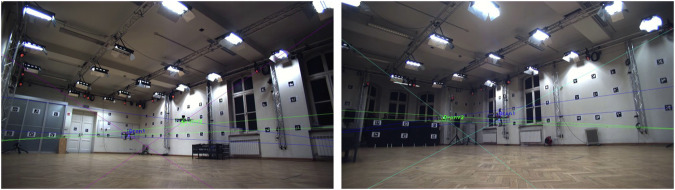
Visualization of the epipolar lines for drones detected on other cameras. The line colors correspond to the cameras.

There are two more issues to be addressed. In the case of the camera model that takes into account distortions, the image coordinates (*x*, *y*) have to be updated according to the taken model. Moreover, the described procedure works only in the case of a single drone flying. If there are more of them, matching of detected drones across cameras is necessary. It can be accomplished based on the epipolar geometry. In such an approach, epipolar lines representing the expected localizations in separate cameras of detected drones on a given one are determined as visualized in Fig. 8. Finally, the matching is conducted to minimize the total distance between epipolar lines and corresponding drones.

#### Particle Swarm Optimization

Obtaining the 3D location of drones can also be achieved by using optimization algorithms and image processing methods. As an example, the method proposed in the paper^21^ can be given, in which a Particle Swarm Optimization (PSO) algorithm^22^, data from multiple cameras, and image processing methods to extract the drone from the image are used. The PSO is a well-known optimization method that has many applications e.g. in signal and image processing, robotics, as well as in problems related to motion tracking^23,24^. The principle of operation of the PSO algorithm is based on a set of particles, each of which represents a hypothetical solution. In the optimization process, particles explore the search space to find the optimal solution to the problem. The best solution is selected based on the fitness function.

In the proposed solution, the particles contain the position in 3D space of all drones on the scene, and the fitness function determines the degree of similarity between the solutions proposed by the algorithm and the actual position of the drones on the scene. In the fitness function, the hypothetical positions of drones, generated by the PSO algorithm, are projected into 2D image space. Next, the bounding boxes approximating the size of the drones in the image from a given camera are determined. Having the bounding boxes representing the drones and the extracted earlier silhouettes of the real drones, their degree of overlap is calculated. Finally, the average overlap value for all drones in the image is established, and the result is averaged over all cameras. For drone silhouette extraction the background/foreground detection algorithm developed by Zivkovic and van der Heijden^25^ was used.

### Inside-Out camera

#### ArUco markers

A precise ArUco marker’s corner localization is essential for camera position and orientation estimation purposes. The more markers are detected and correctly recognized, the more points are available for computing the extrinsic parameters of the camera fixed to the drone: rotation matrix and translation vector, which are then used to determine the current position and orientation of the camera. A predefined set of markers is called a dictionary. In the experiments, markers from the “4 × 4_1000” dictionary were used: 41 markers with identifiers 1-41 to generate simulation data and 83 markers with identifiers 1-83 to generate real data.

The algorithm for processing a video sequence recorded by an FPV camera fixed to the drone’s body to determine the current position and orientation of the drone, which is represented by the current position and orientation of the camera, is as follows:

select the ArUco marker dictionary used in the experiment;

define the internal parameters of the camera;

for each video frame {

convert the image from RGB to monochrome;

detect and recognize ArUco markers in the image, rejecting objects ...

incorrectly recognized as markers;

if at least 4 ArUco markers are detected and recognized, then

determine the current position and orientation of the drone ...

using all the recognized markers;

otherwise

it is impossible to determine the camera position ...

for the current frame;

}

To detect and recognize ArUco markers, the “detectMarkers” function from the ArUco OpenCV library based on the algorithm proposed by Garrido-Jurado *et al*.^26^ was used. The perspective-three-point (P3P) algorithm^27^ is the basis for the “estworldpose” MATLAB function, which returns the pose of a calibrated camera in a world coordinate system as a “rigidtform3d” MATLAB object. The orientation and location of the camera were then extracted from the “R” and the “Translation” properties of the object.

## Technical Validation

The dataset was used for YOLOv5 training and 3D tracking in three different variants.

### Deep neural network training

**Fig. 9:**
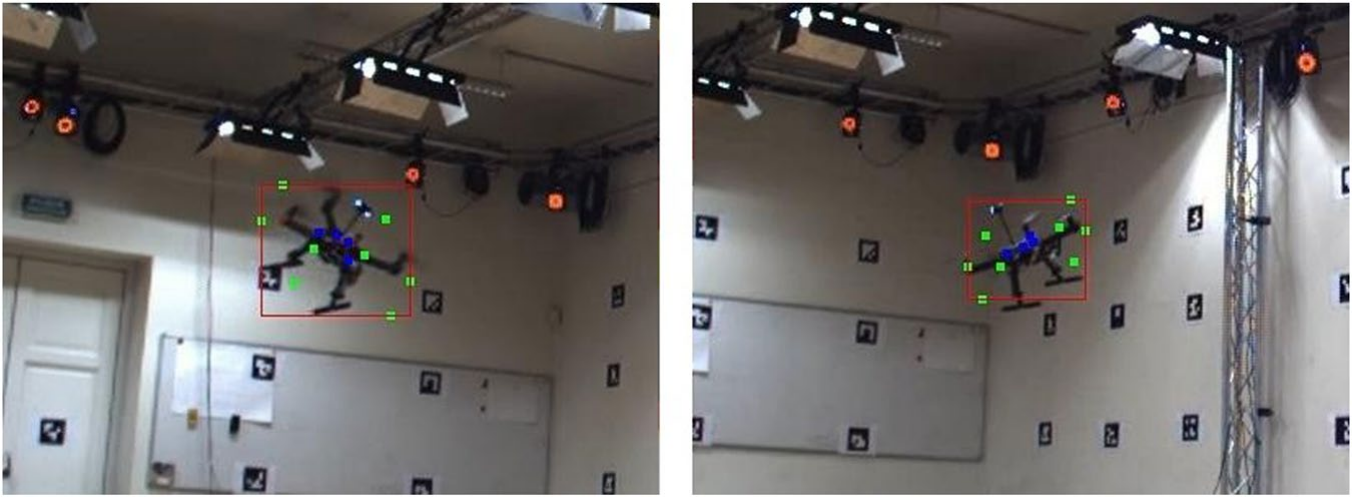
Bounding box extraction based on the motion capture measurements. Markers, 2D and 3D bounding boxes are labeled with blue, green, and red colors, respectively^19^.

Preparing a comprehensive dataset is one of the most important elements needed to train a neural network. Often, real-world datasets require manual annotation of each image. In this case, the Vicon motion capture system was used for this task to automate this process. It includes a set of infrared cameras and four synchronized RGB cameras. To determine the 3D position of the drones, markers were placed on them forming asymmetrical crosses (Fig. 4). By analyzing the mocap data, it was possible to calculate the 3D bounding boxes visualized in Fig. 9. Then the collected data allowed for projecting it into 2D and determining the bounding boxes, which was necessary to create a dataset used to train the neural network. A detailed description of the method, along with trained model detection results, is described by Lindenheim-Locher, W. *et al*.^19^.

### Triangulation based on projection lines

On the registered video sequences, drones are detected by the trained YOLOv5 network. As described previously, it allows for 3D reconstruction. Its performance is assessed by the comparison with ground truth data, using mean as well as median distances selected as measures. Due to possible inaccurate YOLO detections, occlusion and limited field of view, 3D tracking may result in undetected drones. They are summarized as false negatives (FNR) additionally normalized by the number of analyzed video frames and the number of drones.

The obtained results are presented in Table 4. In two sequences denoted as R12_D4 and S09_D6, notable mean errors have been observed, signaling an anomaly. It may be caused by the mismatch between the referenced and estimated drones. This phenomenon is frequently observed when drones operate in close proximity and swaps each other. Despite the perturbation introduced, the median values remain at the same level. The swaps took place in the final part of the recorded experiments, which explains the difference between the mean and median values.

**Table 4:** Tracking errors obtained by triangulation based on projection lines.

Sequence	Mean [m]	Std. dev. [m]	Median [m]	Quartile dev. [m]	FNR [%]
R02_D1	0.075	0.042	0.063	0.013	0.00
R04_D2	0.161	0.440	0.148	0.020	0.00
R12_D4	0.325	1.354	0.152	0.280	1.49
R13_D3	0.160	0.818	0.131	0.020	0.27
S01_D2_A	0.169	0.413	0.165	0.011	0.20
S02_D4_A	0.156	0.435	0.123	0.025	6.60
S09_D6	0.685	1.744	0.076	0.634	0.70

### Particle Swarm Optimization

**Fig. 10:**
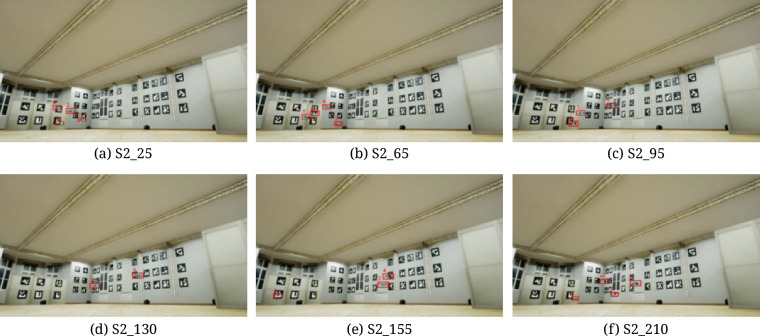
Tracking results for selected frames of sequence S02_D4_A, number of particles: 100, number of iterations: 70.

The method of obtaining the 3D location of drones using the PSO algorithm was tested on three simulation sequences with a different number of drones. The quality of the tracking was evaluated by analyzing qualitative visual assessments and ground truth data. The obtained errors are presented in Table 5, while the example tracking results are shown in Fig. 10. The calculated mean error value and the standard deviation are averaged over 10 runs of the tracking algorithm. Depending on the configuration of the PSO, the average tracking error obtained for individual sequences varies from 0.068 m to 0.510 m. As expected, the best results are achieved with the configuration with the greatest number of particles (100) and iterations (70). The smallest error of 0.068 m was obtained for the S02_D4_A sequence with four drones. For this sequence, the optimal configuration is 50 particles and 70 iterations or 100 particles and 50 iterations. Further reducing the number of particles or iterations causes the tracking error to increase above 0.10 m, which indicates that the small particle population is insufficient for faced demanding 3D tracking problem. Analyzing the results obtained for the S01_D2_A recording, it can be seen that for most configurations, an error of 0.086 or 0.140 m was obtained. There were two drones in this sequence that were tracked well, but at some point, the drones were very close to each other and they were swapped, increasing the tracking error. For the experiments performed, this replacement occurred more often for configurations with a larger number of particles, probably due to the algorithm’s greater coverage of the search space. The worst results are obtained for the S04_D10 sequence (0.193-0.510 m), which is caused by the large number of drones on the scene (10). It is a challenging sequence in which the search space has 30 dimensions, drones often fly close to each other and on the edges of the scene.

**Table 5:** PSO tracking errors for sequences S01_D2_A, S02_D4_A, and S04_D10.

Part	Iter	S01_D2_A		S02_D4_A		S04_D10	
		Mean [m]	Std. dev. [m]	Mean [m]	Std. dev. [m]	Mean [m]	Std. dev. [m]
30	30	0.197	0.217	0.328	0.448	0.510	0.377
30	50	0.086	0.034	0.174	0.257	0.376	0.273
30	70	0.140	0.126	0.137	0.199	0.213	0.147
50	30	0.086	0.034	0.272	0.356	0.392	0.287
50	50	0.086	0.034	0.155	0.209	0.256	0.175
50	70	0.140	0.126	0.090	0.095	0.226	0.165
100	30	0.141	0.126	0.269	0.261	0.260	0.194
100	50	0.140	0.125	0.092	0.099	0.205	0.130
100	70	0.140	0.126	0.068	0.054	0.193	0.118

### ArUco markers

**Fig. 11:**
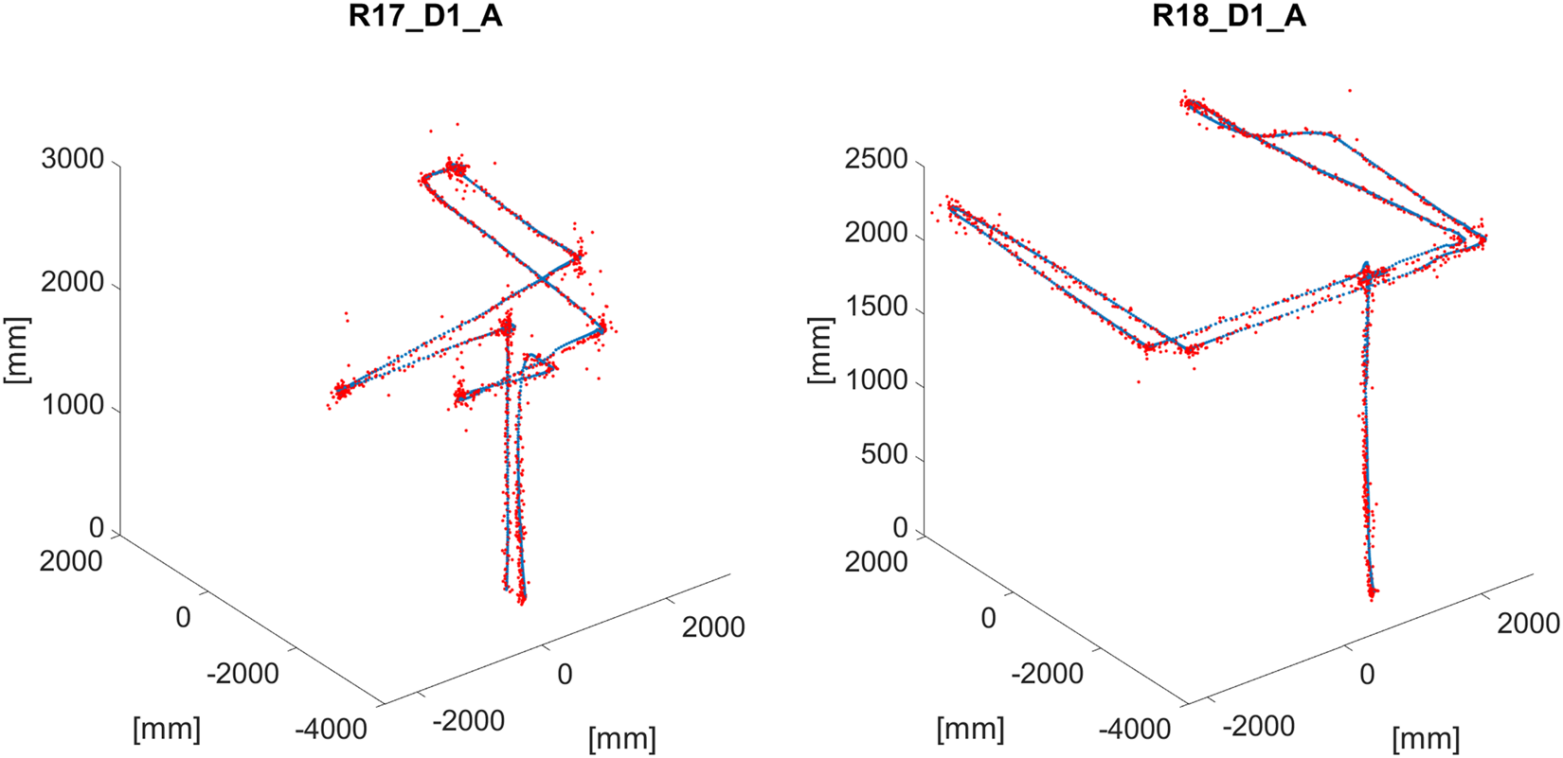
The reconstructed (red) and reference (blue) trajectories for R17_D1_A and R18_D1_A.

A specific feature of the R16_D1_A, R17_D1_A, and R18_D1_A sequences is the FPV recording made by a GoPro camera attached to the drone. The camera’s internal parameter matrix has been created based on the following settings: *FoV*_*x*_ = 92.45°, *FoV*_*y*_ = 60.83° and an image resolution of 3840 × 2160. By placing ArUco markers with known 3D coordinates and identifiers on the walls of the lab in accordance with the prepared plan, it is possible to determine the current position and orientation of the drone in flight and thus recreate step by step the complete flight trajectory. According to the algorithm given in the section “Methods of 3D localization”, trajectories were determined for the R17_D1_A and R18_D1_A sequences (Table 6, Fig. 11), the scenarios of which provided for the following pattern of movements of the drone:

- R18_D1_A - simple maneuvers involving flight at a more or less constant height and overcoming mutually perpendicular sections approximately forming the shape of U in vertical projection,
- R17_D1_A - covering mutually perpendicular sections, combined with making returns and turns.

**Table 6:** Description of the FPV recording.

Sequence	Duration [s]	# of frames with calculated position	# of frames for which position cannot be calculated
R17_D1_A	73.40	1361	475
R18_D1_A	49.24	1218	14

A method has been developed to determine the reference position of the drone using markers on a cross attached to the drone, the positions of which are registered by the Vicon system using the motion capture technique. Orientation reference data were obtained from the GoPro camera’s IMU sensor (recorded at 25 Hz).

Under the assumption that a single frame of the sequence is described by the quaternion *q* = *w* + *xi* + *yj* + *zk*, a rotation matrix can be derived from the quaternion *q* to represent the same rotation. The MATLAB function “quat2rotm” was used for this purpose.

Since the recording of the video sequence by the camera on the drone and the registration of the movement of reference markers by the Vicon system were carried out independently and not synchronized with each other, the comparison of both trajectories - the reconstructed and the reference ones - required determining a common starting point taking into account the difference between the recording frequencies (sequence video - 25 Hz, motion capture technique - 100 Hz). A common starting point also had to be indicated for the orientation data.

After taking into account the difference in registration frequency (every fourth point of the reference trajectory, selected with a step of 40 ms, corresponds to a single point of the reconstructed trajectory), by applying the starting point of the reconstructed trajectory to subsequent points of the initial fragment of the reference trajectory (which was longer), the distance between the corresponding points of both trajectories was calculated along the entire length of the shorter one (i.e., the reconstructed trajectory). The minimum average distance between the corresponding points of both trajectories determined the offset between the initial points of both trajectories, thus minimizing the tracking error.

The reconstructed trajectory may contain incorrect positions, as indicated by, for example, high standard deviation values, and the reason for their occurrence may be the identification of an object incorrectly considered to be an ArUco marker. The problem of assessing the current position and orientation on the fly (in real time) and immediately rejecting erroneous values will be addressed in another work.

The tracking error was determined for a single video frame as the distance between a pair of corresponding points - the point of the reference trajectory and the point of the reconstructed trajectory (Table 7). All ArUco markers recognized in a given frame participated in determining the latter. The accuracy of the reconstructed orientation was assessed by calculating the differences in the roll, pitch, and yaw angles of the reconstructed and reference orientations for the corresponding frames (Table 8).

**Table 7:** The distance between the corresponding points of the reconstructed trajectory and the reference trajectory.

Sequence	Mean [m]	Std. dev. [m]	Median [m]	Quartile dev. [m]
R17_D1_A	0.054	0.077	0.033	0.019
R18_D1_A	0.042	0.045	0.033	0.015

**Table 8:** The difference between the reconstructed orientation and the reference orientation for corresponding frames.

Sequence & angle	Mean [deg]	Std. dev. [deg]	Median [deg]	Quartile dev. [deg]
R17_D1_A roll	6.58	2.39	7.47	0.94
R17_D1_A pitch	7.69	19.14	4.95	0.62
R17_D1_A yaw	6.08	20.14	2.83	1.71
R18_D1_A roll	4.42	3.24	3.99	2.91
R18_D1_A pitch	0.92	0.43	0.86	0.32
R18_D1_A yaw	3.93	2.19	3.88	1.91

## Usage Notes

The presented DPJAIT dataset is a collection of sequences that contain drone flights. Its main application is 3D drone tracking research, with several unique features that will accelerate the development of such methods. As far as we know, this is the only dataset that contains: 1) multi-drone sequences recorded from multiple cameras in the scene; 2) both real and simulation data; 3) recordings from the external system and the drone’s on-board camera; 4) additional ArUco markers placed on the stage. These features enable the dataset to be used in a wide range of problems related to drone position estimation, e.g., 2D and 3D tracking of single and multiple drones, drone location, drone detection, and training deep neural networks. The data package is available for use under the CC BY license and provided by the ZENODO repository^18^.

## Data Availability

An example of the code to generate drone simulations is provided as part of the DPJAIT dataset^18^. The package is composed of two main parts: Unreal 4.27 project with AirSim 1.5.0 plugin and Python scripts, which are used to acquire data, preprocess data, and control simulation. To run the code, two files are needed: an AirSim settings JSON file, which defines simulation objects, and a file, which contains the flight path for the drones. The published package contains both files that were used in the generation of the sequence S13_D3. The manual is included in the readme file.
